# Comparison of outcomes between tubular microdiscectomy and conventional microdiscectomy for lumbar disc herniation: a systematic review and meta-analysis of randomized controlled trials

**DOI:** 10.1186/s13018-023-03962-8

**Published:** 2023-07-03

**Authors:** Tingxin Zhang, Nana Guo, Kaifeng Wang, Gang Gao, Yanhong Li, Feng Gao, Wupeng Yang, Yonghua Wang, Yongjiang Wang

**Affiliations:** 1 Department of Orthopedics, Ordos Central Hospital, 23 Ekin Hollow West Street, Ordos, 017000 China; 2 Critical Care Medicine, Ordos Central Hospital, Ordos, China; 3 Fujian Medical University, Fuzhou, Fujian China

**Keywords:** Tubular microdiscectomy, Lumbar disc herniation, Conventional microdiscectomy, Meta-analysis, Systematic review

## Abstract

**Purpose:**

The clinical outcomes of using a tubular microdiscectomy for lumbar disc herniation were evaluated by comparison with conventional microdiscectomy.

**Methods:**

All of the comparative studies published in the PubMed, Cochrane Library, Medline, Web of Science, and EMBASE databases as of 1 May 2023 were included. All outcomes were analysed using Review Manager 5.4.

**Results:**

This meta-analysis included four randomized controlled studies with a total of 523 patients. The results showed that using tubular microdiscectomy for lumbar disc herniation was more effective than conventional microdiscectomy in improving the Oswestry Disability Index (*P* < 0.05). However, there were no significant differences in operating time, intraoperative blood loss, hospital stay, Visual Analogue Scale, reoperation rate, postoperative recurrence rate, dural tear incidence, and complications rate (all *P* > 0.05) between the tubular microdiscectomy and conventional microdiscectomy groups.

**Conclusions:**

Based on our meta-analysis, it was found that the tubular microdiscectomy group had better outcomes than the conventional microdiscectomy group in terms of Oswestry Disability Index. However, there were no significant differences between the two groups in terms of operating time, intraoperative blood loss, hospital stay, Visual Analogue Scale, reoperation rate, postoperative recurrence rate, dural tear incidence, and complications rate. Current research suggests that tubular microdiscectomy can achieve clinical results similar to those of conventional microdiscectomy.

*PROSPERO registration number* is: CRD42023407995.

## Introduction

Lumbar disc herniation (LDH), namely the degeneration and swelling of the nucleus pulposus of the lumbar intervertebral disc, is one of the most common diseases of the musculoskeletal system. In 1934, Mixter and Barr first described the surgical treatment of LDH [1]. With the continuous progress of microsurgery, the surgical techniques of LDH treatment have been developed rapidly. In 1977, Caspar and Yasargil first applied the conventional microdiscectomy (CMD) to the surgical treatment of LDH [2, 3]. This procedure is considered the gold standard for open discectomy due to its better visibility, less invasive, and lower perioperative morbidity [4]. However, this technique requires incision of the midline ligament structure and separation of the tendon of the paraspinous muscle from the spinous process, which can lead to back pain and even spinal instability after surgery. Over time, surgeons are looking for less invasive procedures to improve clinical outcomes. In 1999, Foley and Smith reported microendoscopic discectomy (MED), which used tubular retractors in conjunction with endoscopes [6, 7]. In 2002, Greiner-Perth et al. [8] reported the combination of tubular retractor and microscope, which overcame the limitation of working scope and achieved better visualization effect. Tubular microdiscectomy (TMD) uses a muscle-space approach instead of the traditional subperiosteal muscle dissection, which reduces tissue damage and accelerates recovery. A meta-analyses have demonstrated that TMD for LDH can produce better or similar outcomes than CMD for LDH [9]. However, all previously published meta-analysis studies had significant limitations, including the absence of randomized controlled studies (RCTs). There is still insufficient level-one evidence to prove the proposed advantages of TMD for LDH. Therefore, we reviewed previous RCTs and conducted this meta-analysis to determining the clinical effectiveness of TMD versus CMD for LDH.

## Methods

### Literature search strategy

We performed systematic literature searches in five electronic databases, including PubMed, Cochrane Library, Medline, Web of Science, and EMBASE. We searched using the following combination of MeSH (Medical Subject Heading) terms and free text words: “tubular microdiscectomy”, “microdiscectomy”, “Lumbar disk herniation” and “Minimally invasive”. The search date was from when databases were built to 1 May 2023. We did not restrict searches based on language or publication year. To prevent certain studies from being missed, we manually searched the bibliographies of RCTs, meta-analyses, and systematic reviews.

### Selection of studies

The study inclusion and exclusion processes were divided into two groups. The selection was first based on the title and abstract, and if a decision could not be made from the summary, the full text was retrieved. When there was a disagreement between the two groups, the selection committee was discussed until a consensus was reached.

### Inclusion and exclusion criteria

We included studies that met the following criteria: (1) Included studies were RCTs. (2) A comparative study on the efficacy of TMD and CMD for LDH. (3) The comparison outcomes included at least one of the following: operating time, intraoperative blood loss, length of stay, Visual Analogue Scale (VAS), Oswestry Disability Index (ODI), reoperation rate, postoperative recurrent rate, incidence of dural tears, and complications rate. Studies were excluded according to the following criteria: (1) Editorials, letters, reviews, case reports, and cadaver or animal experiments. (2) The patient was diagnosed with scoliosis, infection or tumour. (3) Studies that did not meet the inclusion criteria. (4) The data of the comparison outcomes could not be extracted.

### Data extraction

Two reviewers used standardized data extraction tables. The extracted data included authors, publication date, title, country, study design, follow-up duration, number of patients, mean age of patients, type of operation, and comparison outcomes. The comparison outcomes included operating time, intraoperative blood loss, length of stay, VAS, ODI, reoperation rate, postoperative recurrent rate, incidence of dural tears, and complications rate. All data were extracted from article texts, tables, and figures. The research author was contacted for missing data or further information. Two reviewers independently extracted the data; differences were resolved through discussion, and a consensus was reached by including third parties. The data extraction outcomes are shown in Table 1.

**Table 1:** Characteristics of included studies

Author (years)	Country	Study type	Number of samples	Gender (male)	Average age	Follow-up (months)	Outcomes
			TMD/CMD	TMD /CMD	TMD /CMD	TMD /CMD	
Ryang (2008) [10]	Germany	RCT	30/30	13/19	38/39	16/16	1–9
Arts (2011) [11]	Netherlands	RCT	166/159	84/88	41.6/41.3	24/24	1–4, 7, 8
Franke (2009) [12]	Germany	RCT	52/48	26/24	44/44	12/12	1, 5, 6–9
Gempt (2013) [13]	Germany	RCT	19/19	13/8	37/37	33/33	4, 5

*Outcomes*: 1. Operating time, 2. Blood loss, 3. Length of stay, 4. Visual Analog Score, 5. Oswestry Disability Index, 6. Reoperation rate, 7. Postoperative recurrent rate, 8. Incidence of dural tears, 9. Complications rate

*TMD* tubular microdiscectomy, *CMD* conventional microdiscectomy, *RCT* Randomized controlled trial

### Data analysis

**Fig. 1:**
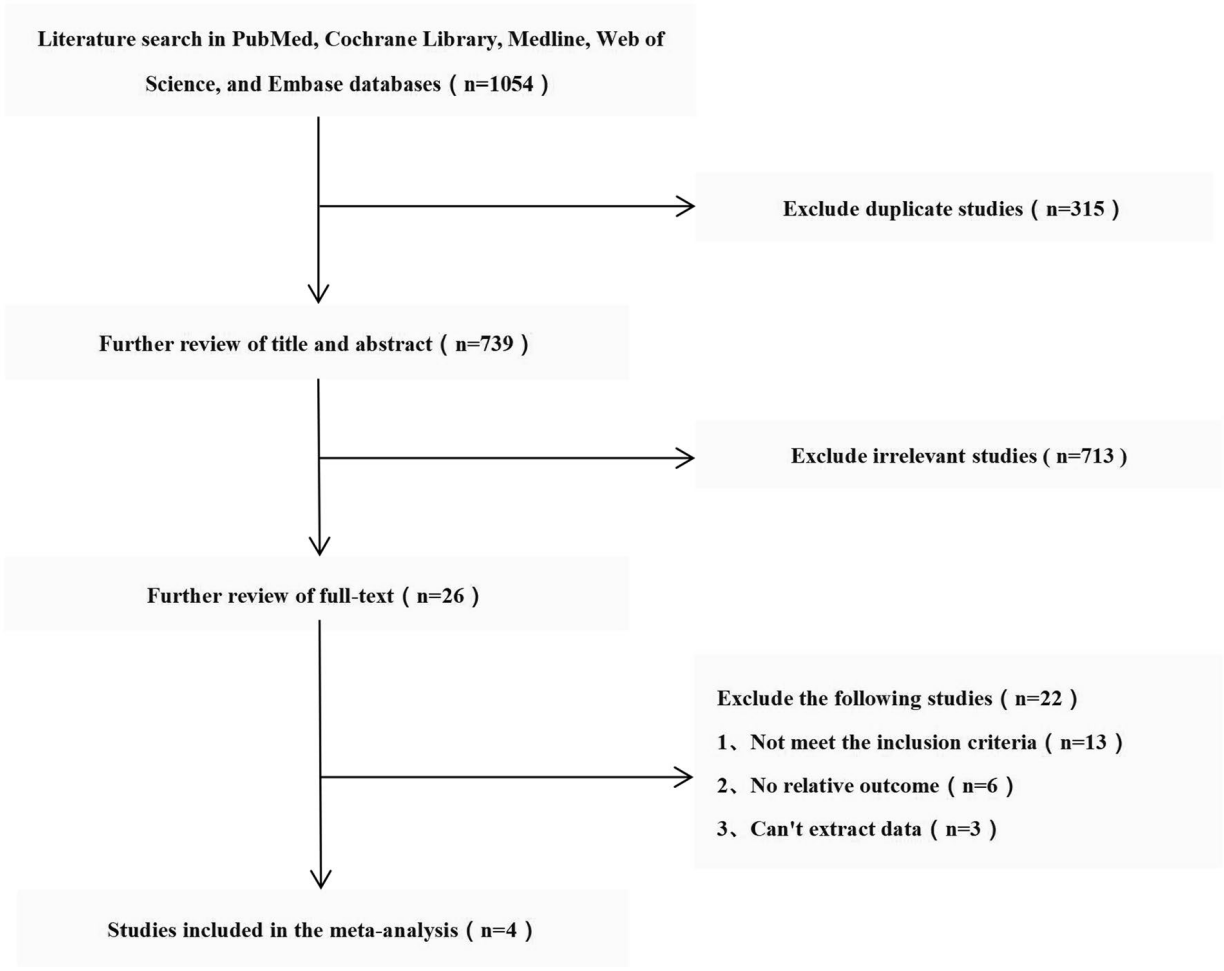
Flow diagram of study selection

We used Review Manager Version 5.4 (Copenhagen: The Nordic Cochrane Centre, The Cochrane Collaboration) to analyse the data of all outcomes and compare the TMD group with the CMD group. For continuous outcomes, such as operating time, length of stay, VAS, and ODI, the means and standard deviations were pooled to a weighted mean difference (WMD) and 95% confidence interval (CI). Risk ratios (RRs) and 95% CIs were used to evaluate dichotomous outcomes, such as reoperation rate, postoperative recurrent rate, incidence of dural tears, and complications rate. We used *I*^2^ to quantify heterogeneity. If *I*^2^ > 50%, the heterogeneity was significant, and the unstandardized mean difference was estimated using a random effects model. Otherwise, a fixed-effects model was applied (Fig. 1).

### Quality assessment

**Fig. 2:**
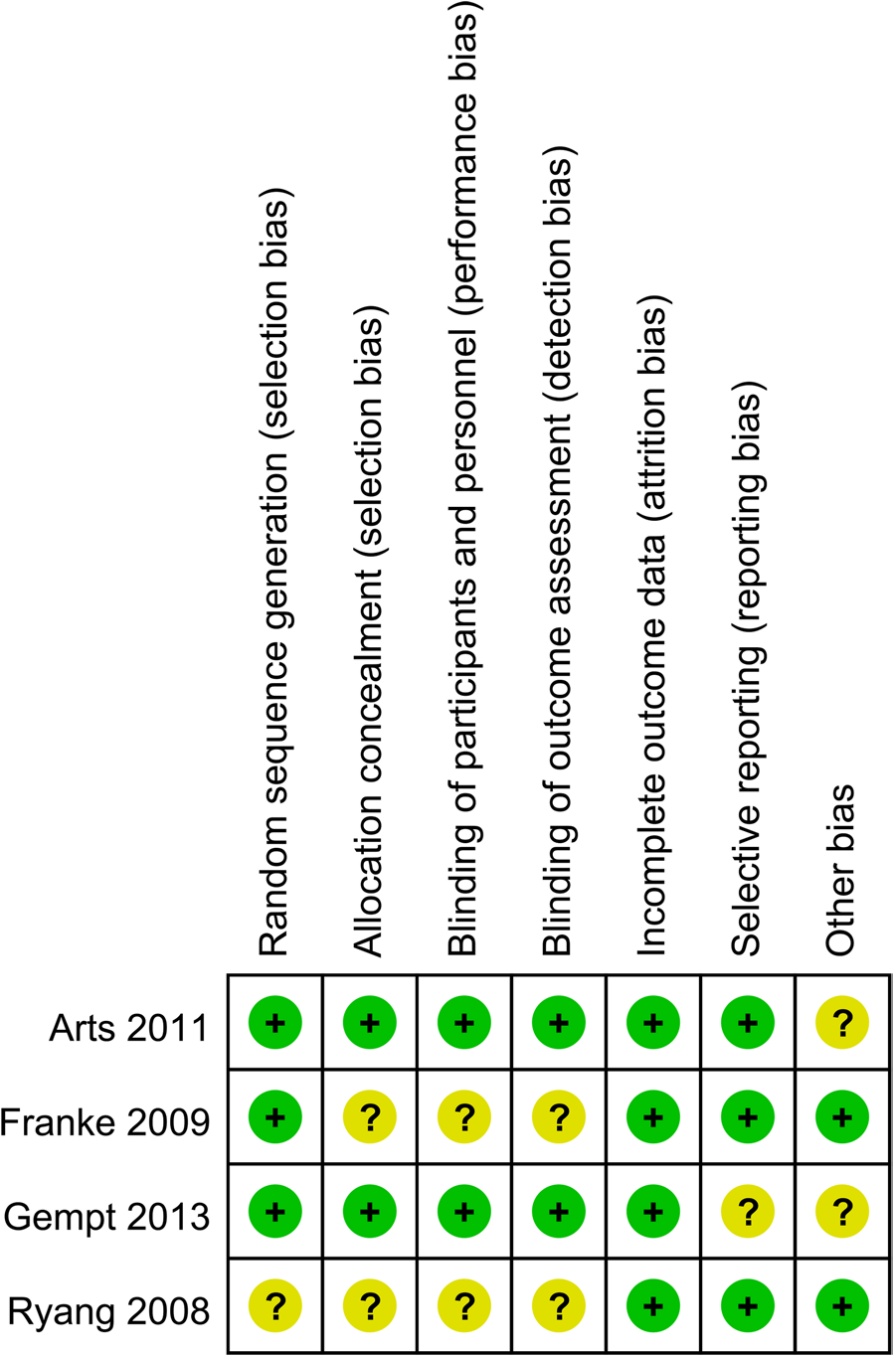
The methodological quality of the randomized controlled trials

For RCTs, the Cochrane Handbook for Systematic Reviews of Interventions was used [27], including 7 domains: random sequence generation, allocation concealment, blinding of participants and personnel, blinding of outcome assessment, incomplete outcome data, selective outcome reporting, and other sources of bias (Fig. 2). Two reviewers independently carried out the quality assessment and discussed disagreements with a third party.

### Literature search

There were 1054 studies identified from five electronic databases (Fig. 1). Of those, 315 studies were duplicates, and 713 studies were excluded after title and abstract screening. After careful full-text evaluation, 4 studies [10–13] were reviewed, and the data were extracted. The demographic and clinical characteristics of the 4 studies are described in Table 1. A total of 267 patients who underwent TMD were compared with 256 patients who underwent CMD. The mean follow-up time was more than 12 months, and the mean age of the patients was 37–44 years. Operating times were reported for 3 studies [10–12]. Intraoperative blood loss was reported for 2 studies [10, 11]. Length of stay was reported for 2 studies [10, 11]. VAS was reported in 3 studies [10, 11, 13]. ODI were reported in 3 studies [10, 12, 13]. Reoperation rate, postoperative recurrent rate, incidence of dural tears, and complications rate were reported in 3 studies [10–12].

### Operating time

**Fig. 3:**
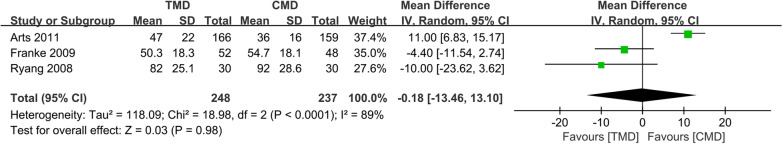
Meta-analysis of TMD group versus CMD group in operating time

Three studies [10–12] with 248 and 237 patients compared the mean operating time between the TMD and CMD groups. The meta-analysis indicated no significant differences between the TMD and CMD groups (WMD − 0.18; 95% CI − 13.46 to 13.10; *P* > 0.05). The heterogeneity test outcome (*I*^2^ = 89%) indicated significant heterogeneity (Fig. 3).

### Length of stay

**Fig. 4:**
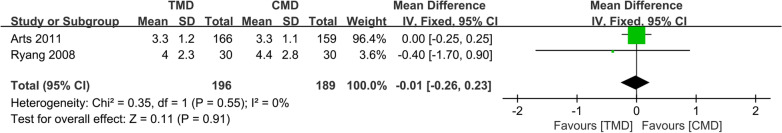
Meta-analysis of TMD group versus CMD group in length of stay

Three studies [10, 11] with 196 and 189 patients compared the mean length of stay between the TMD and CMD groups. The meta-analysis indicated no significant differences between the TMD and CMD groups (WMD − 0.01; 95% CI − 0.26 to 0.23; *P* > 0.05). The heterogeneity test outcome was *I*^2^ = 0%, and the fixed-effects model was applied (Fig. 4).

### VAS

**Fig. 5:**
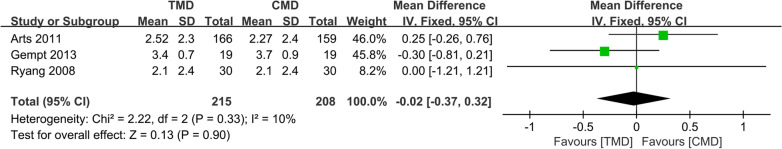
Meta-analysis of TMD group versus CMD group in VAS

Two studies [10, 11, 13] with 215 and 208 patients compared the mean VAS between the TMD and CMD groups. The meta-analysis indicated no significant differences between the TMD and CMD groups (WMD − 0.02; 95% CI − 0.37 to 0.32; *P* > 0.05). The heterogeneity test outcome was *I*^2^ = 10%, and the fixed-effects model was applied (Fig. 5).

### ODI

**Fig. 6:**
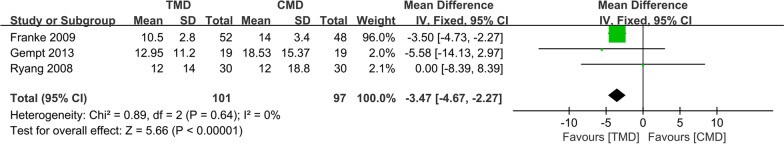
Meta-analysis of TMD group versus CMD group in ODI

Three studies [10, 12, 13] with 101 and 97 patients compared the mean ODI between the TMD and CMD groups. The meta-analysis indicated no significant differences between the TMD and CMD groups (WMD − 3.47; 95% CI − 4.67 to 2.27; *P* > 0.05). The heterogeneity test outcome was *I*^2^ = 0%, and the fixed-effects model was applied (Fig. 6).

### Reoperation rate

**Fig. 7:**
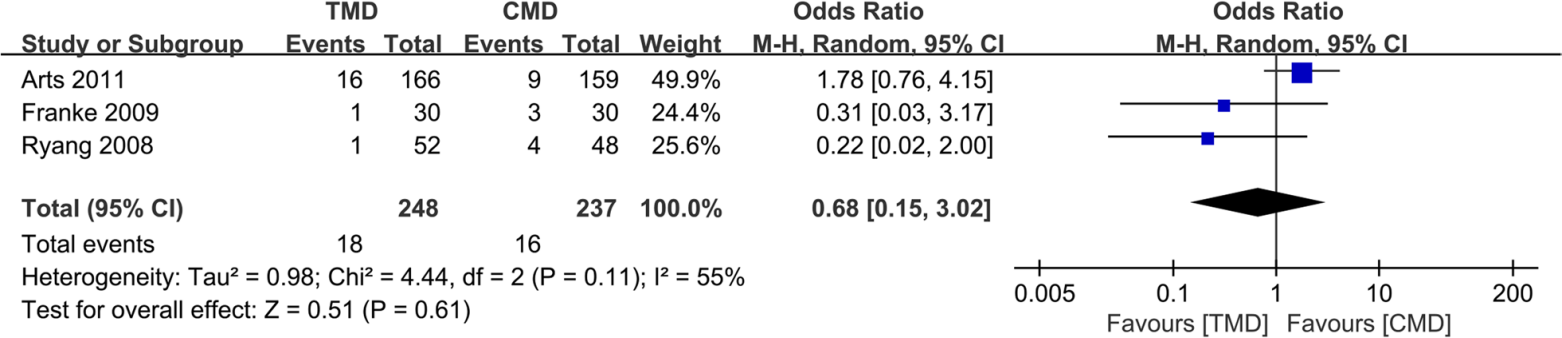
Meta-analysis of TMD group versus CMD group in reoperation rate

Three studies [10–12] with 248 and 237 patients, respectively, compared the reoperation rate between the TMD and CMD groups. The pooled outcomes indicated no significant differences between the TMD and CMD groups (RR 1.32; 95% CI 0.75 to 2.31; *P* > 0.05). The heterogeneity test outcome was *I*^2^ = 0, and the fixed-effects model was applied (Fig. 7).

### Incidence of dural tears

**Fig. 8:**
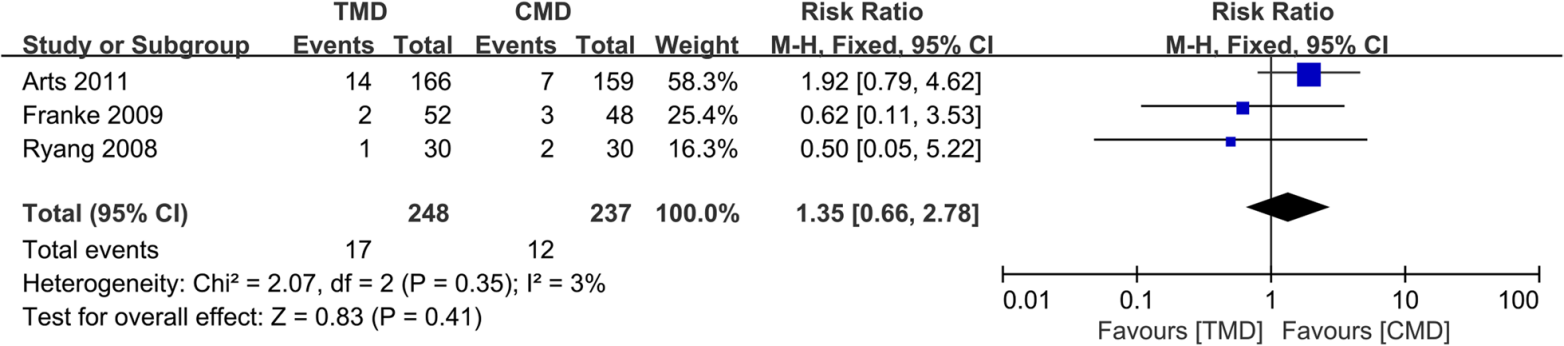
Meta-analysis of TMD group versus CMD group in incidence of dural tears

Three studies [10–12] with 248 and 237 patients, respectively, compared the incidence of dural tears between the TMD and CMD groups. The pooled outcomes indicated no significant differences between the TMD and CMD groups (RR 1.35; 95% CI 0.66 to 2.78; *P* > 0.05). The heterogeneity test outcome was *I*^2^ = 3, and the fixed-effects model was applied (Fig. 8).

### Postoperative recurrent rate

**Fig. 9:**
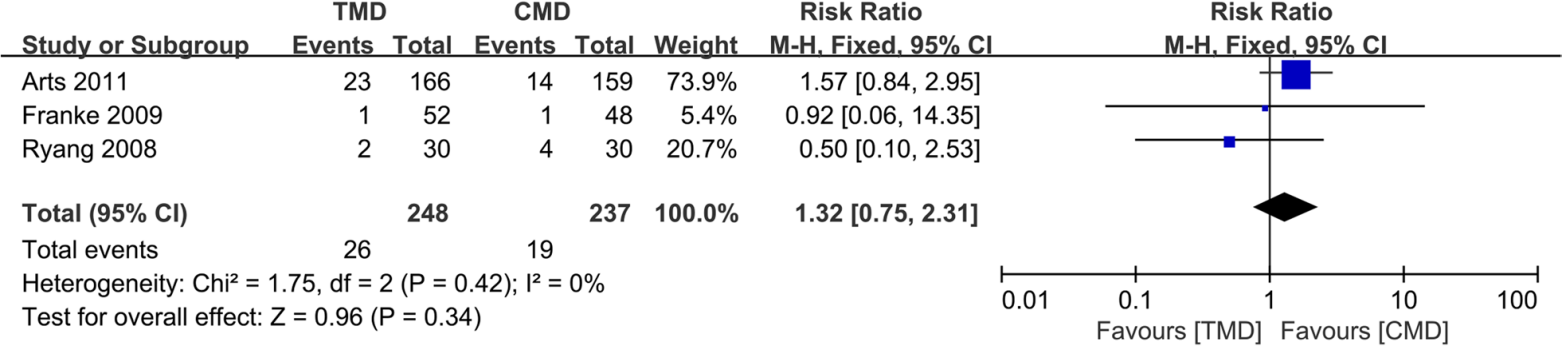
Meta-analysis of TMD group versus CMD group in postoperative recurrent rate

Three studies [10–12] with 248 and 237 patients, respectively, compared the postoperative recurrent rate between the TMD and CMD groups. The pooled outcomes indicated that no significant differences between the TMD and CMD groups (RR 0.69; 95% CI 0.17 to 2.85; *P* > 0.05). The heterogeneity test outcome (*I*^2^ = 54%) indicated significant heterogeneity (Fig. 9).

### Complications rate

**Fig. 10:**
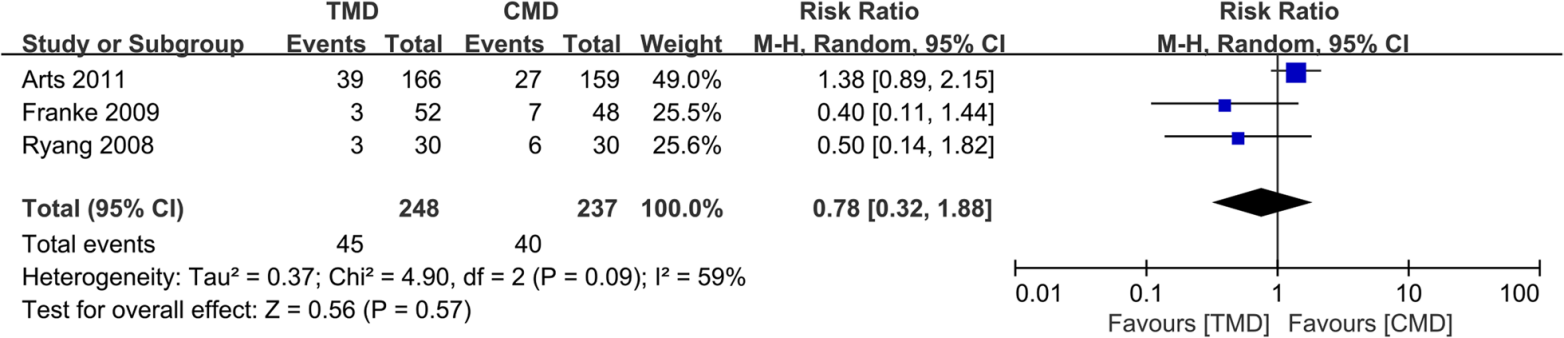
Meta-analysis of TMD group versus CMD group in complications rate

Three studies [10–12] with 248 and 237 patients, respectively, compared the complications rate between the TMD and CMD groups. The pooled outcomes indicated that no significant differences between the TMD and CMD groups (RR 0.78; 95% CI 0.32 to 1.88; *P* > 0.05). The heterogeneity test outcome (*I*^2^ = 59%) indicated significant heterogeneity (Fig. 10).

## Discussion

Surgery of LDH is one of the most common neurosurgical procedures. Currently, CMD is the gold standard for surgical treatment of lumbar disc-related sciatica [14]. The advent of microsurgery has led to the development of novel surgical techniques for addressing LDH [15]. Similar to other surgical fields, there have been various endeavours to apply endoscopic techniques to spinal surgery. However, these attempts have not been widely accepted thus far [16]. The use of endoscopes for spine surgery has become more common, but the limitations of the two-dimensional image produced by the endoscope have become more apparent. To address the issue of limited access and visibility in surgical procedures, a new approach has been developed that combines tubular retractors and trocar systems with the aid of a microscope [17].

Despite the potential benefits of TMD, there is currently insufficient evidence to support the notion that it can produce results comparable to or better than CMD. Li et al. [8] recently published a meta-analysis comparing the clinical efficacy of TMD and CMD. The study found no significant difference in the clinical efficacy between the two treatments. The study's data extraction and analysis were deemed imprecise, and there was a high risk of bias due to the inclusion and pooling of N-RCTs. Therefore, there is currently insufficient evidence to compare the efficacy of TMD with CMD.

In order to assist surgeons in making clinical decisions and developing optimal treatments for LDH, a meta-analysis was conducted to analyse the data of the TMD and CMD. Our study employed a meticulous search strategy, limited to RCTs, and adhered to Cochrane Collaboration-approved systematic review methods. In our meta-analysis, the information was extracted from 4 published RCTs using the Cochrane Handbook for Systematic Reviews of Interventions for quality assessment. The outcomes indicated that the included literature was of high quality. Our study found no statistically significant differences between the TMD and CMD groups in regard to operative time, length of stay, VAS, ODI, reoperation rate, postoperative recurrence rate, dural tear rate, and complication rate.

There was no significant difference in operative time between the two groups. However, it is worth noting that there was considerable heterogeneity, which could be attributed to the varying experience levels of the surgeons [18]. Two studies were conducted to calculate intraoperative blood loss. Arts et al. [11] found that 90% of patients in the TMD group had less than 50 mL intraoperative blood loss, while 85% of patients in the CMD group had the same (*P* < 0.08). Ryang et al. [10] also reported that there was no significant difference in intraoperative blood loss between the TMD group and the CMD group. Our study could not be analysed as specific data on bleeding volume could not be extracted from the two studies mentioned. The functional improvement of a patient can be evaluated using the ODI score and VAS score. It has been observed that TMD, which involves intermuscular approach, is less invasive than CMD, which involves subperiosteal paraspinal muscle dissection [19]. As a result, postoperative low back pain and leg pain may be less severe in TMD than in CMD. However, previous studies have yielded different results. Overdevest et al. [20] reported that the clinical outcomes of TMD compared with CMD were not statistically significant. Our study did not find a significant difference between the TMD and CMD groups in terms of VAS scores. However, when it comes to ODI, the combined result favoured the TMD group (*P* < 0.05).

In theory, TMD overcomes the two-dimensional problem and obtains better visualization effects [21, 22], which will reduce the occurrence of complications to a certain extent. However, our study results indicate that there was no significant difference in terms of dural tear rate and complication rate between the TMD and CMD groups. The incidence of dural tear was 7% in the TMD group and 5% in the CMD group, with no significant difference between the two groups. The authors suggest that this could be due to better visualization of cerebrospinal fluid leaks under the microscope. Therefore, TMD can be considered as safe as CMD in terms of surgical outcomes. According to Ostermann's [23] report on 35,309 patients over 11 years, the reoperation rate after lumbar disc surgery was 14%. Several studies [24–26] have been conducted with a follow-up period ranging from 4 to 10 years, indicating that the recurrence rate of lumbar disc herniation ranges from 5 to 19%. Our pooled results indicate that the reoperation rate in the TMD group was 10.5% compared to 8% in the CMD group, but the difference between the two groups was not statistically significant.

One limitation of this paper is the limited sample size of our study. While all included studies were randomized controlled trials, the study scale was small and the total sample size was low. Additionally, the age and gender distribution of patients, various indications for surgery, experience level of orthopedic surgeons, and severity of LDH were not consistent across the original studies.

## Conclusion

According to our meta-analysis, the TMD group outperformed the CMD group in terms of ODI score. However, there were no significant differences between the two groups in terms of operating time, intraoperative blood loss, hospital stay, VAS, reoperation rate, postoperative recurrence rate, dural tear incidence, and complications rate. According to current research, TMD can produce clinical results that are similar to those of CMD. However, further prospective studies with a larger sample size are required to confirm whether TMD is a superior alternative to CMD.

## Data Availability

All data generated or analysed during this study are included in this published article and its supplementary information files.

## Abbreviations

LDH: Lumbar disc herniation
TMD: Tubular microdiscectomy
CMD: Conventional microdiscectomy
ODI: Oswestry Disability Index
VAS: Visual Analog Scale
RCTs: Randomized controlled trials
WMD: Weighted mean difference
CI: Confidence interval
RRs: Risk ratios

## References

[1] Mixter WJ, Barr JS (1934). Rupture of intervertebral disc with involvement of the spinal canal. N Engl J Med.

[2] Caspar W (1977). A new surgical procedure for lumbar disc herniation causing less tissue damage through a microsurgical approach.

[3] Yasargil MG, Wullenweber R, Brock M, Hamer J (1977). Microsurgical operation of herniated disc. Advances in neurosurgery.

[4] Riesenburger RI, David CA (2006). Lumbar microdiscectomy and microendoscopic discectomy. Minim Invasive Ther Allied Technol.

[5] Katayama Y, Matsuyama Y, Yoshihara H, Sakai Y, Nakamura H, Nakashima S, Ito Z, Ishiguro N (2006). Comparison of surgical outcomes between macro discectomy and micro discectomy for lumbar disc herniation: a prospective randomized study with surgery performed by the same spine surgeon. J Spinal Disord Tech.

[6] Foley K (1997). Microendoscopic discectomy. Tech Neurosurg.

[7] Smith MM, Foley KT (1998). Microendoscopic discectomy (MED): the first 100 cases. Neurosurgery.

[8] Greiner-Perth R, Böhm H, El Saghir H (2002). Microscopically assisted percutaneous nucleotomy, an alternative minimally invasive procedure for the operative treatment of lumbar disc herniation: preliminary results. Neurosurg Rev.

[9] Li X, Chang H, Meng X (2018). Tubular microscopes discectomy versus conventional microdiscectomy for treating lumbar disk herniation: systematic review and meta-analysis. Medicine (Baltimore).

[10] Ryang YM, Oertel MF, Mayfrank L, Gilsbach JM, Rohde V (2008). Standard open microdiscectomy versus minimal access trocar microdiscectomy: results of a prospective randomized study. Neurosurgery.

[11] Anderson PA (2010). Tubular discectomy resulted in greater leg and back pain and a lower rate of recovery than conventional microdiscectomy for sciatica. J Bone Joint Surg Am.

[12] Franke J, Greiner-Perth R, Boehm H, Mahlfeld K, Grasshoff H, Allam Y, Awiszus F (2009). Comparison of a minimally invasive procedure versus standard microscopic discotomy: a prospective randomised controlled clinical trial. Eur Spine J.

[13] Gempt J, Jonek M, Ringel F, Preuss A, Wolf P, Ryang Y (2013). Long-term follow-up of standard microdiscectomy versus minimal access surgery for lumbar disc herniations. Acta Neurochir (Wien).

[14] Peul WC, van Houwelingen HC, van den Hout WB, Brand R, Eekhof JA, Tans JT, Thomeer RT, Koes BW (2007). Leiden-The Hague Spine Intervention Prognostic Study Group. Surgery versus prolonged conservative treatment for sciatica. N Engl J Med.

[15] Williams RW (1978). Microlumbar discectomy: a conservative surgical approach to the virgin herniated lumbar disc. Spine.

[16] Schreiber A, Suezawa Y (1986). Transdiscoscopic percutaneous nucleotomy in disk herniation. Orthop Rev.

[17] Brock M, Kunkel P, Papavero L (2008). Lumbar microdiscectomy: subperiosteal versus transmuscular approach and influence on the early postoperative analgesic consumption. Eur Spine J.

[18] Parikh K, Tomasino A, Knopman J, Boockvar J, Härtl R (2008). Operative results and learning curve: microscope-assisted tubular microsurgery for 1- and 2-level discectomies and laminectomies. Neurosurg Focus.

[19] German JW, Adamo MA, Hoppenot RG, Blossom JH, Nagle HA (2008). Perioperative results following lumbar discectomy: comparison of minimally invasive discectomy and standard microdiscectomy. Neurosurg Focus.

[20] Overdevest GM, Peul WC, Brand R, Koes BW, Bartels RH, Tan WF, Arts MP (2017). Leiden-The Hague Spine Intervention Prognostic Study Group. Tubular discectomy versus conventional microdiscectomy for the treatment of lumbar disc herniation: long-term results of a randomised controlled trial. J Neurol Neurosurg Psychiatry.

[21] Gibson JN, Waddell G (2007). Surgical interventions for lumbar disc prolapse: updated Cochrane Review. Spine.

[22] Teli M, Lovi A, Brayda-Bruno M, Zagra A, Corriero A, Giudici F, Minoia L (2010). Higher risk of dural tears and recurrent herniation with lumbar micro-endoscopic discectomy. Eur Spine J.

[23] Osterman H, Sund R, Seitsalo S, Keskimäki I (2003). Risk of multiple reoperations after lumbar discectomy: a population-based study. Spine.

[24] Bruske-Hohlfeld I, Merritt JL, Onofrio BM, Stonnington HH, Offord KP, Bergstralh EJ, Beard CM, Melton LJ, Kurland LT (1990). Incidence of lumbar disc surgery. A population-based study in Olmsted County, Minnesota, 1950–1979. Spine.

[25] Keskimäki I, Seitsalo S, Osterman H, Rissanen P (2000). Reoperations after lumbar disc surgery: a population-based study of regional and interspecialty variations. Spine.

[26] Malter AD, McNeney B, Loeser JD, Deyo RA (1998). 5-year reoperation rates after different types of lumbar spine surgery. Spine.

[27] Higgins JP, Altman DG, Gøtzsche PC, Jüni P, Moher D, Oxman AD, Savovic J, Schulz KF, Weeks L, Sterne JA (2011). The Cochrane Collaboration’s tool for assessing risk of bias in randomised trials. BMJ.

